# Policy interventions for improving hospital-to-home transitions of care for older adults and informal caregivers: a qualitative study

**DOI:** 10.1186/s13584-025-00692-6

**Published:** 2025-06-06

**Authors:** Opeyemi Rashidat Kolade, Joshua Porat-Dahlerbruch, Theo van Achterberg, Moriah Esther Ellen

**Affiliations:** 1 Department of Health Policy and Management, Guilford Glazer Faculty of Business and Management and Faculty of Health Sciences, Ben-Gurion University of the Negev, David Ben Gurion Blvd 1, Be’er Sheva, Israel; 2 Department of Public Health and Primary Care, Academic Centre for Nursing and Midwifery, University of Leuven, KU Leuven, Kapucijnenvoer 7, 3000 Leuven, Belgium; 3 Department of Acute and Tertiary Care, School of Nursing, University of Pittsburgh, 336 Victoria Building, 3500 Victoria Street, Pittsburgh, PA 15261 USA; 4 Institute of Health Policy Management and Evaluation, Dalla Lana School of Public Health, University of Toronto, Health Sciences Bldg, 155 College Street, Toronto, ON M5T 3M7 Canada

**Keywords:** Hospital-to-home transitions, Older adults, Caregivers, Policy, Qualitative

## Abstract

**Background:**

Efficient hospital-to-home transitions for older adults and their informal caregivers are hampered by current fragmented care systems, resulting in communication and coordination lapses when people move between hospital-to-home settings. Such fragmentation often leads to suboptimal hand-overs of information and care, medication errors, and overlooked follow-up appointments, which, in turn, contribute to adverse health outcomes for the elderly population. This study aims to answer the question: “What policy interventions can improve the transitions from hospital to home for older adults and their informal caregivers” Thus the study focuses on delineating policy recommendations at the micro, meso, and macro levels to facilitate smoother and more beneficial hospital-to-home transitions for older adults and their informal caregivers.

**Methods:**

As part of the European Union Transitional Care Program (TRANS-SENIOR), this qualitative descriptive study leverages a multiple perspectives approach through in-depth interviews with older adults and informal caregivers. The goal is to pinpoint critical intervention zones of policy recommendations based on a holistic understanding of older adult and caregiver recommendations for improving hospital-to-home transitions.

**Results:**

Findings show strategies that strengthen patient and caregiver engagement on the micro level. These include implementing personalized care plans and improving communication channels between healthcare providers and their recipients. The meso level targets healthcare organizations and systems, promoting the adoption of streamlined care coordination, enhanced discharge planning, and bolstered support services for caregivers. Such interventions are designed to smooth the transition process, ensuring that care continues seamlessly from hospital to home. At the macro level, our findings urge policy reforms to address broader systemic issues, such as the allocation of resources, the introduction of funding mechanisms, and the expansion of healthcare workforce capacity. These policy recommendations aim to create an enabling environment for effective care transitions, addressing underlying challenges that impede seamless care transitions.

**Conclusion:**

This paper presents a set of policy recommendations for policymakers, healthcare professionals, and stakeholders. These recommendations aim to tackle the multifaceted challenges associated with hospital-to-home transitions to enhance care experience and outcomes for older adults and their caregivers by addressing individual, organizational, and systemic issues.

**Supplementary Information:**

The online version contains supplementary material available at 10.1186/s13584-025-00692-6.

## Background

In Israel and worldwide, health systems are being challenged by the increasing number of older people requiring hospital admissions [1, 2]. It is becoming more challenging to meet the complex care needs of older adults and prepare them and their informal caregivers for the transition home. As a result, older adults and their caregivers are being sent home in increasingly frail states, with little support and a high risk for adverse events leading to early readmission [1, 3–5]. Furthermore, inadequate care coordination and person-centered attention, bureaucratic hurdles in receiving post-discharge care services, and caregiver burden and responsibility of care after discharge are other challenges encountered by older adults and informal caregivers [6]. These challenges highlight the need for policy interventions to improve hospital-to-home transitions for older adults and their caregivers. This work, which is within TRANS-SENIOR, an innovative research network focusing on care transitions, aims to delineate policy recommendations at the micro, meso, and macro levels to facilitate smoother and more beneficial hospital-to-home transitions for Israeli older adults and their informal caregivers. Our findings will inform policy development, clinical practice, and community initiatives to optimize the well-being and outcomes of older adults and their informal caregivers during this critical period. 

### Literature review: transitional care

Extensive interventions have been implemented to improve hospital discharge for older adults. This has led to numerous complex interventions, often targeting different aspects and levels of the healthcare system [7–15]. Although the interventions vary, a common trend in newer initiatives is the focus on policy changes, multiple layers of interventions, or new models of care delivery, with the belief that to create real and sustainable change, it is essential to alter the structures and processes of care delivery [16, 17].

Furthermore, transitional care, which is the care around the movement of patients between care settings and providers [18], is influenced by various factors, such as personal, local, organizational, and policy contexts [6]. For example, a study by Fakha and colleagues [19] revealed that implementing transitional care interventions in Leuven, Belgium, was impacted by organizational factors, with individual engagement and strategic partnerships playing key roles. Naylor and Berlinger [20] emphasized that organizational, regulatory, financial, and cultural barriers hinder the widespread adoption of evidence-based transitional care. Weeks and colleagues [21] identified program scope, structure, continuity of care, funding, and health system infrastructure as critical factors influencing the development and success of transitional care programs in Canada. Kansagara and colleagues [22] highlighted the necessity for further research to identify effective strategies for improving care transitions at national, regional, and local health systems levels. Similarly, studies have emphasized the need to seize context-specific opportunities to optimize care transitions at organizational and individual levels [19]. These studies collectively underscore the importance of considering broader and holistic contexts, e.g., local, organizational, and policy contexts, when designing and implementing transitional care interventions.

Finally, hospital-to-home transitions are often poorly managed, leading to a higher incidence of adverse events and avoidable rehospitalizations [9, 23, 24]. Studies emphasize the importance of well-prepared discharges and appropriate transitional care in reducing the likelihood of adverse health outcomes in the initial weeks post-discharge [25]. Hospital-initiated transitional care interventions, particularly those incorporating predischarge and post-discharge interventions, have been shown to reduce readmission and emergency department visit rates [26]. Older adults often feel overwhelmed by the sudden shift to self-care, while informal caregivers experience high-stress levels and insufficient preparation [27, 28]. Effective collaboration between older adults, caregivers, and healthcare providers is essential during these transitions, with support teams playing a crucial role in managing health, mobility, and engagement at home [6]. Implementing patient-centered care, improving communication between healthcare providers and patients, enhancing coordination of healthcare services, and ensuring the availability of necessary resources are vital steps to support a well-managed transition from hospital to home, ultimately optimizing health outcomes and reducing healthcare costs for older adults and their caregivers [24].

In Israel, research on transitional care interventions has been limited [1]. While previous studies within the Israeli context have highlighted the importance of optimizing post-discharge nutritional management for older adults with poor socio-economic status [29], the importance of telephone follow-ups following hospital discharge [30], the association between readmission risks and post- discharge Primary Care Physicians’ (PCP) review of the discharge summary [31], and culturally tailored transitional care [32–34], our study offers a unique contribution to advancing transitional care practices and policies in Israel. Our study emphasizes the needs of older adults and their informal caregivers, addressing a critical population group that faces distinct challenges during care transitions. Furthermore, by addressing micro (individual and caregiver), meso (organizational), and macro (policy and systemic) levels, the study provides a multi-level approach to understanding and improving care transitions. Our study thus aims to delineate policy recommendations at the micro, meso, and macro levels to facilitate smoother and more beneficial hospital-to-home transitions for older adults and their informal caregivers in Israel.

## Methodology

### Study design

This study employed a multiple-perspective descriptive, qualitative approach using in-depth interviews with two groups of service users: older adults and informal caregivers. This methodology synthesizes policy interventions at multiple levels: macro, meso, and micro levels [35, 36] by analyzing similarities in viewpoints from multiple perspectives. Our conceptual lens is based on an implementation science view of systems of macro-, meso-, and micro-level actors affecting patient outcomes [35, 37].We sought to answer the research question: “What policy interventions can improve the transitions from hospital to home for older adults and their informal caregivers?” Initial policy interventions were synthesized. Converging, complementary, and diverging patterns between participant groups were identified using the guide by Vogl and colleagues [38]. As analysis progressed, the initial list of policy interventions was updated accordingly, leading to the development of a final set of policy interventions across macro, meso, and micro health system domains.

#### Participants and recruitment

This study focused on recruiting recently discharged older adults aged 65 years and older and informal caregivers of older adults who had recently been discharged from the hospital following any cause of admission. To capture a nationwide perspective, we aimed to recruit participants from different regions within Israel representing peripheral and central healthcare services. The main inclusion criteria were that older adults (65 +) had to have recently (within the last year) experienced returning home after hospital discharge, and informal caregivers had to have cared for their loved ones who recently (within the previous year) experienced hospital-to-home transitions. The informal caregivers were recruited based on their caregiving roles in supporting older adults, rather than being a dyadic recruitment of older adult and the informal caregiver of the recruited older adult.

For recruitment, we employed a marketing research agency with expertise and a database of target audiences based on their previous recruitment for other research purposes and access to diverse recruitment channels within Israel. The recruitment process was conducted impartially and ethically. A questionnaire focusing on characteristics such as hospital discharge within the last year, supporting an older family member through a recent discharge, and socio-demographic characteristics such as age, region, employment status, and educational status was sent to a pool of older adults and informal caregiver participants. Participants who met the recruitment criteria were contacted and recruited over 3 months. Recruitment involved contacting individuals via telephone calls and emails. Interviews were scheduled with participants who consented to participate. 

#### Data collection

Data were collected using a semi-structured, one-to-one interview guide. The guide was adapted from McMaster University’s citizen panel question guide for involving older adults with complex care needs and informal caregivers to improve hospital-to-home transitions [39]. All research team members approved of the questions. One-on-one interviews allowed participants to freely express their answers to the questions with a sense of privacy [40]. Data were collected both in Hebrew and English. Participants who spoke Hebrew were interviewed by a trained and experienced staff from the market research agency. A research team member interviewed English-speaking participants. The interviews took place on Zoom between November 2023 and January 2024. Interviews lasted for an hour on average. Interview questions and probes included demographic characteristics of participants and suggested policy interventions to enhance transitions from hospital to home at individual, organizational, and policy levels (Box 1). Probing and clarifying questions were asked in addition to these main questions to clarify participants’ responses. Recordings were transcribed, de-identified, and stored on a secured server.

**Table:** 

Interview questions
Micro level
1. What role would you (and your caregiver) like to play in your own care during hospital to- home transitions?
2. What supports would enable you (and your caregiver) to play that role?
Meso level
3. What could providers do individually and as a team to improve the quality of transitions of care?
4. What role would you (and your caregiver) like to play in this process?
5. What supports would enable you (and your caregiver) to play that role?
Macro level
6. What could decision-makers do to improve the quality of hospital-to-home transitions?
7. What role would you (and your caregiver) like to play in this process?
8. What supports would enable you (and your caregiver) to play that role?
9. How will we know if the quality of hospital-to-home transitions has improved?

### Data analysis

Thematic analysis was conducted to identify commonalities and essential patterns of meaning relating to our specific research objective of proposing policy recommendations [41]. We used an inductive, data-driven approach to understand participants’ perspectives regarding the research objective [42]. Initial codes were generated from recurring statements in the interviews and then organized into themes reflecting the policy interventions on the three interest levels. Relevant quotations were selected to substantiate these themes. During the analysis, the research team utilized peer debriefing to ensure that preconceived codes did not constrain the data. Instead, the coding process was iterative, with the coding structure being adjusted as the analysis progressed. One researcher independently developed the initial code list. A second team member supervised the data analysis process closely, reviewed all codes and the coding process, providing feedback on the initial code lists. Regular meetings involving all team members facilitated in-depth engagement with the data, review of developed codes, and discussion of emerging codes and themes [41]. The research team analyzed the data and grouped it into policy intervention categories with descriptions for all categories. We then compared the data between older adults and informal caregivers (participant triangulation) [43]. Participant triangulation elucidates converging, diverging, and complementary patterns between participant groups [38, 43]. Converging, diverging**,** and complementary patterns for each policy intervention category were recorded. The categories and descriptions were adjusted based on observed patterns. This process continued until the research team agreed on the final list of intervention categories, organized into macro, meso, and micro domains.

### Trustworthiness

We used Guba’s recommendations to ensure trustworthiness and rigor [44]. For example, by cross-verifying data interpretation among research team members. To enhance credibility, we used participant and investigator triangulation [43] and provided direct quotes exemplifying the policy interventions [45]. We verified accurate transcriptions and maintained an audit trail throughout the analysis, which enhanced the dependability [45]. Peer debriefing facilitated confirmability. Transferability was enhanced by describing the methods and participant characteristics, which the audience can use to determine the applicability of results to their setting. The reporting of this study complies with the Standards for Reporting Qualitative Research (SRQR) [46] (Additional file 1).

### Ethics approval and participant consent

This study was approved by the Ben-Gurion University Human Subjects Research Committee (ME30032003). All participants provided written informed consent before participating in the study.

## Results

There were 16 participants in this study: seven older adults and nine informal caregivers. The mean age of older adults was 69, with the oldest participant being 76, while informal caregivers had an average age of 53. There were more female caregivers (n = 7) than male caregivers (n = 2). The older adult male-to-female ratio was fairly equal, 4:3, and so was the English-to-Hebrew speaking participant ratio: 8:8 for both groups of older adults and informal caregivers. Participants were recruited from central Israel and peripheral regions (north and south). Most informal caregivers were university degree holders and were gainfully employed (full or part-time). The relationships recruited caregivers shared with their older adults were child (n = 5), spouse (n = 2), grandchild (n = 1), and in-law (n = 1). The reasons for hospitalization in both groups ranged from cardiovascular, neurological, respiratory, orthopedic, and gastrointestinal issues. More participants (three older adults and six informal caregivers) were admitted to government hospitals as opposed to private (n = 1) and HMO-owned hospitals (n = 6). In terms of hospital size, only two older adult participants were from small hospitals (0–300 beds). Table 1 displays the demographic characteristics of participants per participant category.

**Table 1:** Characteristics of participants

Participant category		N = 16
	Older adult (n = 7)	Informal caregiver (n = 9)
*Gender*		
Male	4	2
Female	3	7
Mean age (years)	69	53
Duration of hospital stay (range in days)	3–7	2–30
*Region*		
Central & Tel-Aviv	5	5
Jerusalem	1	0
Southern	1	1
Northern	0	3
*Education*		
High school/Postsecondary	3	1
University	4	8
*Employment status*		
Working for pay (full/part-time)	3	7
Retired	4	2
*Hospital type*		
Government	3	6
Private	1	0
HMO (Clalit, Maccabi)	1,2	3,0
*Hospital size (number of beds)*		
Large (≥ 800)	4	3
Medium (300–800)	1	6
Small (0–300)	2	0

### Policy intervention categories

**Fig. 1:**
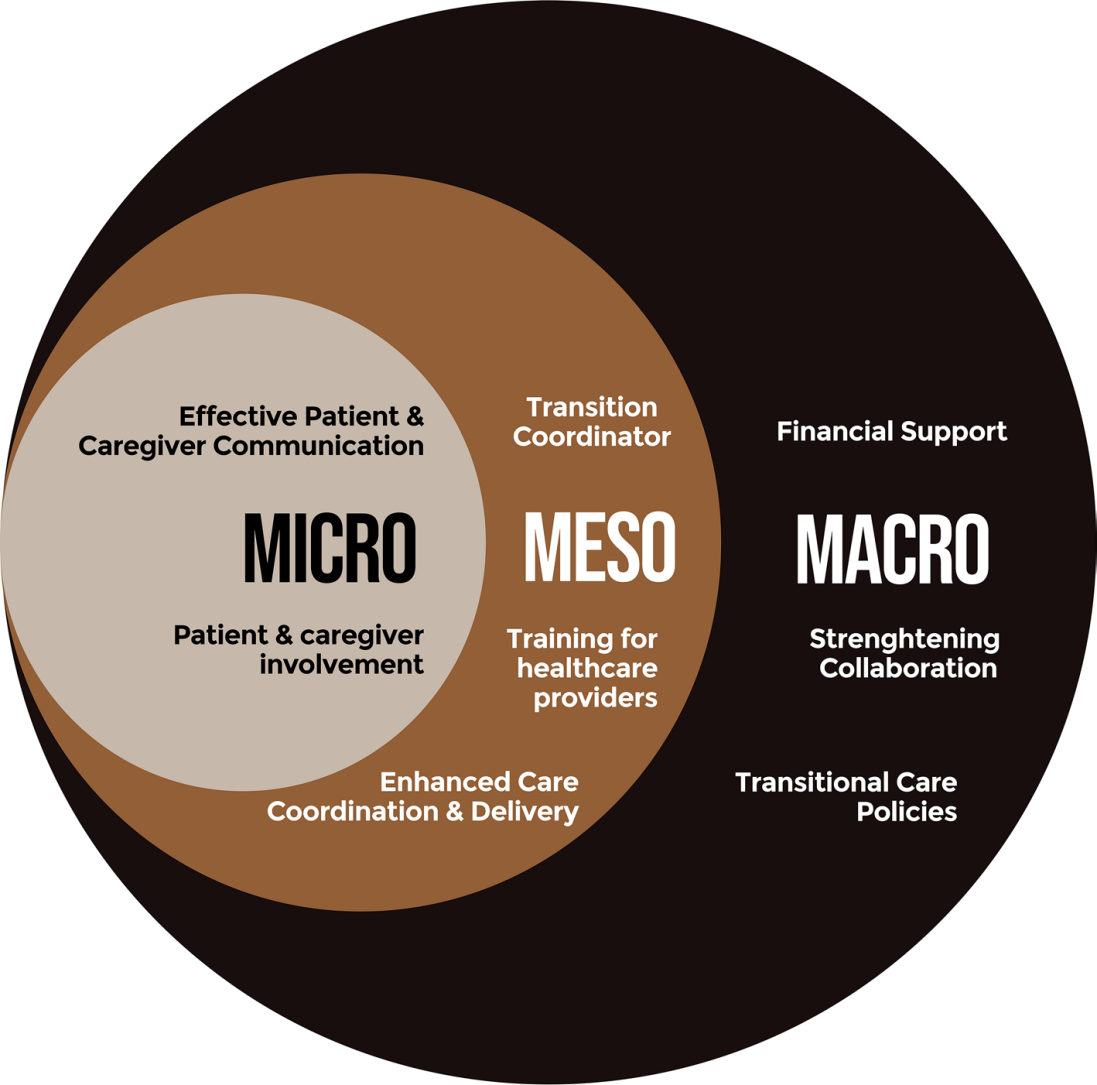
Overview of policy intervention categories. *Source* Authors’ analysis. This overview categorizes policy interventions for improving the transition experiences from hospital to home for older adults and their informal caregivers. The domains are macro, i.e., national or jurisdictional policies; meso, i.e., care organizations, e.g., Hospitals or Health Maintenance Organizations (HMOs); and micro levels, i.e., individual units and care teams). Each level is vital in supporting smoother transitions for older adults and their caregivers to improve hospital-to-home transition experiences of older adults and informal caregivers

The policy intervention categories are illustrated in Fig. 1; summary descriptions for each policy intervention can be found in Table 2. Each ring represents one of the three health system levels at which policy interventions would influence improving the experiences of hospital-to-home transitions for older adults and informal caregivers. The macro-level intervention groups are financial support, strengthening collaboration, and developing and implementing supportive transitional care policies. The meso-level intervention categories are to designate a transition coordinator, care coordination and delivery, and training for healthcare providers. Finally, the micro-level intervention categories are effective patient and provider communication and patient and caregiver involvement.

**Table 2:** Policy interventions for enhancing hospital-to-home care transitions for older adults and informal caregivers

Policy categories	Description
	Macro: Nation, federal ministries, National Insurance Institute
Financial support from the government and NII	Increase financial assistance for vulnerable elderly Incorporate relevant post-discharge services in the NII basket e.g., for emotional/psychological therapy
Strengthening collaboration	Improve coordination between hospitals, social workers, municipal authorities, healthcare providers, and government agencies Improve access to necessary medications, equipment, and supportive post-discharge care at home Increase collaboration between healthcare institutions and patients to implement supportive measures
Developing and promoting supportive transitional care policies	Support employers to allow employees more time to care for their loved ones Provide options for financial support and direct caregiving assistance for vulnerable older adults Provide emotional therapy/support to address the psychological impact of dependency and significant health events
	Meso: Hospitals, Health Maintenance Organizations (HMOs)
Designating a transition coordinator	Appoint a contact person to facilitate communication between patients and healthcare providers, ensuring continuity of care and addressing post-discharge needs
Enhanced care coordination and delivery	Implement a more integrated system for appointment scheduling to reduce wait times Streamline the discharge process Provide comprehensive post-discharge support, including coordination of follow-up appointments and medication management Promote a cross-sectoral approach to improve transition/post-discharge services
Training for healthcare providers	Increase personalized attention and empathy in healthcare delivery, Train healthcare professionals to adopt a more patient-centered approach Enhance healthcare professionals’ training to emphasize empathy, communication skills, and patient-centered care
	Micro: Unit, care team
Effective patient and provider communication	Prioritize patient-centered care; schedule dedicated time for discharge discussions to avoid last-minute briefings Ensure both older adults and informal caregivers are present during key information exchanges Facilitate effective communication in healthcare decision-making processes Using plain language and visual aids where necessary, provide clear explanations and discharge instructions to older adults and informal caregivers Adopt structured communication protocols, e.g., the Teach-Back Method, to confirm understanding of medications and discharge plans Emphasize the importance of compassionate and friendly interactions between hospital staff and patients Promote an environment of respect and dignity by encouraging staff to introduce themselves clearly, maintain eye contact, and take time to listen attentively Use patient and caregiver satisfaction surveys to evaluate care transition services and to inform improvement initiatives
Patient and caregiver involvement	Actively engage older adults and informal caregivers in care transitions, such as hospital discharges, involve them in planning meetings, explaining next steps in care, and identifying support needs Encourage healthcare teams to assess and document older adult and caregiver preferences, needs, and goals in the care process, updating them regularly throughout hospitalization and transitions

Descriptions of the interventions were derived from the fragments coded to identify policy intervention categories. In the text, we include these descriptions and direct quotes that illustrate the policy intervention categories in each domain. The descriptions also explain where the opinions of different participant groups aligned and where they diverged

### Macro level

#### Financial support

Many older adults and informal caregiver participants emphasized the need for government support, mainly through financial assistance for families dealing with healthcare expenses, especially vulnerable elderly individuals. Respondents highlighted the financial strain of purchasing expensive medications and treatments, often without adequate government subsidies. They recommended providing more comprehensive healthcare coverage, including support for alternative therapies like acupuncture and natural remedies.

*“I wish the government was more supporting financially because these families paying a lot of money for a lot of things…we didn’t have any medical insurance, like private insurance, to cover all these expenses.”* ~ *Informal caregiver*

*“My case might not be the most representative, given that I am relatively youthful at 69 and retain considerable physical strength. However, individuals in the 75–80 age bracket necessitate increased attention and support, especially as their financial conditions may not always be conducive to self-sufficiency.”* ~ *Older adult*

They described the need for a more comprehensive support system for older adults undergoing significant health challenges, particularly those who require caregiving assistance post-discharge or face functional limitations. They highlighted the importance of National Insurance or social workers aiding and guiding older adults and informal caregivers during the transitional phase, ensuring that patients feel supported and cared for in their homes.

*“While we are financially secure and do not face issues in that regard, for individuals reliant solely on National Insurance, the presence and guidance of a social worker during this transitional phase are crucial.”* ~ *Older adult*

Additionally, there were recommendations for emotional therapy or support to address the psychological impact of dependency and significant health events. According to participants, suddenly moving from being an independent and healthy person post-discharge to constantly being dependent on a caregiver for activities of daily living was “depressing.” Thus, additional emotional support was recommended to enhance hospital-to-home transitions and improve overall health outcomes.

*“It won’t hurt to provide some emotional therapy after surgery. After all, this is an independent adult who suddenly becomes dependent, and emotionally that is harmful that suddenly you depend on someone else, and that dependency makes you depressed”* ~ *Older adult*

#### Strengthening collaboration

Participants emphasized that improved coordination between hospitals and communities is essential for enhancing hospital-to-home transitions. This involves hospitals working closely with community healthcare providers, social services, and support organizations to create a seamless care continuum. This approach includes timely communication of patient information, coordinated care plans, and follow-up support to address each patient’s unique needs. Moreover, community-based resources can offer ongoing support, education, and assistance, helping patients and their families manage health conditions effectively at home. In summary, both groups of participants called for improved coordination and strengthened collaborations between hospitals, healthcare providers, and government agencies to ensure smoother transitions for patients after hospital discharge, including access to necessary medications, equipment, and supportive care at home. Participants’ recommendations included the need for hospitals and municipal authorities to communicate regarding discharged patients’ status.

*“Hospitals should communicate with municipal authorities regarding the status of discharged patients, including financial means, health status, and specific needs. Yes, this should be executed through collaboration between hospital social workers and their municipal counterparts.”* ~ *Older adult*

These recommendations underscore the importance of collaboration between various stakeholders, including government agencies, healthcare providers, patients, and community support services, to enhance the quality and accessibility of care for individuals in need.

#### Developing and implementing supportive transitional care policies

Participants also recommended that the government develop and promote policies beneficial for older adults and informal caregivers’ transition experience and overall outcomes. A recurring example of an area for fostering favorable policies was identifying caregivers’ roles in the care transition process and providing employment benefits for working informal caregivers and respite care for them. In the same vein, providing options between direct caregiving and financial support was emphasized. Both participant groups agreed that the older adults’ and their caregivers’ values and preferences should be considered regarding what support they prefer: visiting a rehabilitation facility or returning to their homes to recover fully in a familiar environment and with care from their loved one.

*“I think it should be an option. If somebody wants to take time out from work to do that, so they should help them financially to do that.”* ~ *Informal caregiver*

*“Following a cerebral incident three years ago, I was presented with two options: either to receive partial financial support for caregiving or to be provided with a caregiver in lieu of financial assistance. A similar model could be beneficial in the current context, potentially alleviating the strain on both my family and me.”* ~ *Older adult*

Both participant groups recommended a policy intervention focused on providing some form of respite for family caregivers if they choose to support their vulnerable older adult transitioning home from the hospital. Both participant groups agreed on developing and implementing policies that can significantly improve care transition experiences. They also proposed a government intervention to support employers in allowing employees more time to care for their loved ones, indicating a collaborative effort between the government and private sectors.

### Meso level

#### Transition coordinator

At the organizational, hospital, and Health Maintenance Organization (HMO) levels, participants proposed policy interventions for improved care transition and older adult support after discharge from the hospital. They recommended the implementation of a designated transition coordinator or contact person to facilitate communication between patients and healthcare providers, ensuring continuity of care and addressing post-discharge needs. This role would involve following up with discharged patients, providing guidance, and offering emotional support during the challenging transition.

*“…like the secretary of the department or a designated person that is the transition coordinator, and that is her job is to call all the patients that were discharged and to make sure that they are okay. Because it is a very confusing time when you’re discharged, and yeah, it is a little traumatic; it is traumatic.”* ~ *Older adult*

The recommendation to implement a transition coordinator role was a key priority across all participant groups. Participants viewed this role as vital for ensuring person-centered care and maintaining continuity of care. This was especially important for chronically ill patients, such as older adults with heart failure or cancer.

*“Heart failure is a fast heart rate and difficulty breathing, and every action makes it hard to breathe and gives you heart palpitations. I would like more personal attention. If the hospital had the financial ability to provide a dedicated person who could guide each patient, that would be the best.”* ~ *Older adult*

#### Training for healthcare providers

There was a strong emphasis on the importance of personalized attention and empathy in healthcare delivery. Healthcare professionals were recommended to receive training to adopt a more patient-centered approach, focusing on empathy and communication skills. This training should create a more compassionate and effective healthcare environment.

*“Maybe like take them for … courses of how to address differently and to get like a wake-up call that, okay, in front of you, somebody is sitting, a mother, a father, a sister.”* ~ *Informal caregiver*

#### Enhanced care coordination and delivery

The solutions proposed by the participants aim to enhance the experiences of older adults and their informal caregivers. These interventions include developing a more integrated system for scheduling follow-up appointments. Streamlining the discharge process and providing comprehensive post-discharge support —such as coordinating follow-up appointments and managing medications— were also recommended. These policy interventions seek to address organizational-level inefficiencies and ultimately improve healthcare outcomes for individuals within the healthcare system. These policy interventions.

*“A decision should be made to streamline the process for patient discharge from the hospital…For example, my 90-year-old father frequently requires hospital visits and finds the necessity of multiple doctors’ appointments burdensome.”* ~ *caregiver*

Streamlining the discharge process and providing comprehensive post-discharge support, including coordination of follow-up appointments and medication management, can alleviate patient anxiety and improve continuity of care.

*“Implementing a more integrated system shouldn’t pose significant challenges. Considering the amount of time I spent navigating various medical professionals, doctors, nurses, cardiologists, family doctors, and other specialists if there had been one person to coordinate my treatment plan and check the progress every few months, it would have significantly alleviated my anxiety. It would have been more cost-effective for the healthcare system and more convenient and beneficial for my well-being.”* ~ *Older adult*

*“I believe the issue is not one of financial constraints but rather a lack of openness or decisiveness in policy. A decision should be made to streamline the process for patients discharged from the hospital, as many are in similar situations. For example, my 90-year-old father frequently requires hospital visits and finds the necessity of multiple doctor appointments burdensome.”* ~ *Informal caregiver*

### Micro level

#### Effective patient and caregiver communication

Participants emphasized the importance of patient-centered care and effective communication in healthcare decision-making processes. Patients and their families value being involved in treatment and discharge plan decisions. Hence, they emphasized the significance of clear explanations from healthcare providers and the importance of compassionate and friendly interactions between hospital staff and older adults/informal caregivers.

*“For a nurse to come and have a final talk before the discharge and say"[Participant’s name], your mother is feeling better, and there is currently nothing we can do for her; every day she stays here, she risks further infections, so it’s best if she continues her treatment from home. We will, of course, be in contact with you daily; we will send a nurse from the hospital/HMO after 48 hours to give her blood tests, check her blood pressure, see that her blood is flowing properly, that her heartbeat is normal, give her an EKG..."* ~ *Informal caregiver*

#### Patient and caregiver involvement

Patient feedback was emphasized as essential, suggesting collaboration between healthcare providers and patients to improve service delivery. Further emphasizing strengthening collaborations between hospitals and communities, participants highlighted the General Practitioners’ role in constantly engaging, especially with older adult patients and their caregivers. Table 3 presents additional quotes from the interviews by themes.

## Discussion

This study identified policy interventions for enhancing hospital-to-home care transitions for older adults and informal caregivers. It delineates policy recommendations for improving transitional care experiences for older adults and informal caregivers on three comprehensive levels: micro, meso, and macro. We used a multi-perspective approach to gain a complete patient perspective of recommendations to improve hospital-to-home transitions. Older adults and informal caregivers who are first-hand knowledge holders of the experiences of navigating hospital-to-home care transitions proposed recommendations for addressing challenges encountered during this critical period. The recommendations proposed by older adults and informal caregivers were primarily based on their perspectives rather than those of policymakers or healthcare professionals. Each policy intervention category described in Table 2 is based on research data. The macro-level policy interventions focus on national or government-level policymaking, with recommendations directed at individuals/bodies such as policymakers and government ministries, e.g., Ministries of Health, social welfare, etc. On the other hand, the meso-level policy interventions described in this study target healthcare organizations, Health Maintenance Organizations, and hospital administrations. Finally, micro-level policy interventions target individuals and relationships between healthcare providers and older adults/informal caregivers. Our research findings build on the work of Ellen and colleagues, who revealed that health system policymakers in Israel are open to using evidence-based research in their decision-making processes [47]. This study adds to the existing body of knowledge in transitional care in Israel and health policy development. To the best of our knowledge, no research has previously delineated policy interventions at the three health system levels to guide policy development for enhancing hospital-to-home transitions of care for older adults and informal caregivers. While some previous studies have proposed interventions targeting healthcare providers at the micro and organizational levels [19, 48–50], others have proposed policy initiatives only at the macro level [51]. Yet, some others discuss intervention components from two of the three levels [52]. Our research, however, clearly highlights interventions across multiple levels, emphasizing the importance of the inter-relationship between the three levels, similar to complex adaptive systems [53–55].

Our research findings align with previous findings from the literature regarding proposed solutions and recommendations to improve care transition for the population groups of interest. For example, Boye and colleagues [56] emphasized the importance of continuity of care for acutely hospitalized older multimorbid patients, focusing on improving communication and mutual understanding between patients and healthcare professionals to ensure a seamless experience of hospital-to-home transition. Furthermore, in Israel, Theilla and colleagues emphasized the importance of continuity of care in ensuring optimal outcomes for geriatric patients from lower socio-economic backgrounds [29]. Participants in this current study echoed this micro-level recommendation. Furthermore, along the same lines of effective patient and provider communication for effective hospital-to-home transition experience, it is important to state that for the Israeli context, as well as similar healthcare systems with cultural and linguistic diversities, there is a growing recognition of the need to make services more accessible to cultural and linguistic minorities and studies have been conducted to examine the relationship between the quality of hospital-to-home transitional care and language concordance [32, 57, 58]. For example, In Israel, Ethiopian immigrants have access in some clinics and hospitals to employed facilitators/liaisons, and there are Arab primary care providers (PCPs) in Arab villages. However, when it comes to specialty outpatient care and hospitals, significant cultural and linguistic barriers remain [59, 60].

**Table 3:** Additional quotes from interviews by themes

Policy level	Theme	Example quote
Macro	Financial support	*“My mom wasn’t working that time. But some people are young, and they stopped work, …, we didn’t have any medical (private) insurance to cover all these expenses,… it was hard financially because we did all ourselves. I wish the government would support more with financial,”* ~ *Informal caregiver*
	Strengthening collaboration	*“It’s not just the Ministry of Health, but also the Ministry of [Labor, Social Affairs and] Social Services can help.”* ~ *Informal caregiver*
		*“Following hospitalization, I was deemed stable with a reduced risk of immediate recurrence. I was discharged and advised to seek continued care in the community. However, it was unclear who in the community I should consult. There is a lack of a single entity that can oversee and coordinate my ongoing care. Ideally, I would have preferred a professional to review my medical history and guide me accordingly”* ~ *Older adult*
	Developing and implementing supportive transitional care policies	*“The government can promote certain policies that are good for the people. On one hand, I think the government should be involved in changing the policy, but I think they can help the HMOs to supervise and to get to that solution without being in their business [not interfering directly in the operations of HMOs, rather support and oversee them—helping guide them toward effective solutions that ultimately protect the best interests of patients,]”* ~ *Informal caregiver*
Meso	Transition care coordinator	*“The only thing [is] to have the transition coordinator, she follows up, she will make the phone call, and she’ll make sure that everything goes according to plan and you’re feeling good and everything is okay. you feel like somebody cares about you.”* ~ *Informal caregiver*
		*“[Have] a dedicated nurse in the community who will also call to check in, [e.g.,] a social worker too, especially when she knows it’s an elderly person who lives alone without a carer.”* ~ *Informal caregiver*
	Training for healthcare providers	*“I really think the training… just to see the person as a person and not a patient…training on how to be released [discharged], to give a number,…[so] there’s a contact, we don’t have to tell the whole story again…* ~ *Informal caregiver*
		*“A small-scale workshop for 4–5 patients would be beneficial. This would provide straightforward, simple communication, potentially including insights from a patient who has undergone a similar experience. This approach would be preferable to a purely technical interaction, such as wait for the discharge letter, wait for this, sit here.”* ~ *Older adult*
	Enhanced care coordination and delivery	*“I would like more personal attention. If the hospital had the financial ability to provide a dedicated person who could guide each patient, that would be the best.”* ~ *Informal caregiver*
Micro	Effective patient and caregiver communication	*“…Minimal yet meaningful interaction with a healthcare professional concerned about our well-being, ensuring all needs are met, would have been appreciable. I recognize their time constraints and do not expect extensive care…That is the primary idea that comes to mind.”* ~ *Older adult*
	Patient and caregiver involvement	*“My opinion is that GPs should demonstrate greater engagement, perhaps making monthly inquiries about a patient’s health status to monitor progress. This would be beneficial.”* ~ *Older adult*

Similarly, on the meso level, the recommendation for using transition coordinator support to enhance hospital-to-home care transition was echoed by participants. This aligns with a previously conducted randomized controlled trial to evaluate the impact of a brief nurse practitioner intervention on care transitions among older hospitalized adults discharged to home. A nurse practitioner provided up to three home visits and two telephone calls, including medication review, care coordination, assessment of medical care needs, and brief coaching in self-management skills. Findings suggested that intervening at the point of transition may extend the reach of the primary care physician by improving patient outcomes through nursing support at a high-risk period of the transition from hospital to home [61]. A similar RCT implemented an evidence-based patient-centered intervention of individually tailored care delivered by a registered nurse to older adults with multimorbidity discharged from the hospital to the community reported improvement in patient experience [50]. Finally, our findings on training healthcare providers tie closely with other research [62, 63], and Treister-Goltzman and Peleg’s policy proposal for increasing physician shortage in Israel [64]

Furthermore, financial incentives for improving care transitions across practice settings are nonexistent or, in some situations, run contrary to the needs of the patient [65]. Coleman and colleagues proposed that financial incentives should be established that encourage clinicians in different settings to implement and contribute to a uniform, comprehensive plan of care [65]. In the same vein, similar to our findings centered on funding support from the government on the macro level, Fakha and colleagues identified resource allocation as an important factor imperative for the implementation of transitional care interventions [19].

Recent research confirms the importance of delineating policy interventions into macro, micro, and meso levels with evidence that decisions at one level affect the others. For example, the trickle-down effect of macro-level interventions, either through legislative or regulatory support, on the meso and micro levels [66, 67]. Shaw et al. (2017) explored how macro, meso, and micro-level policies can be intertwined in the hospital-to-home transitions. They emphasized that the vital links among the three levels are not just theoretical but are based on real interpersonal relationships among those who represent these levels: policymakers, organizational leaders, and care providers. Without these relationships, the coordination and effectiveness of care across different levels would be compromised [68]. Furthermore, Fakha and colleagues reported that providing financial resources to support transitional care innovations was an important influencer in implementing other interventions [19]. For this current research, we see the interplay of these relationships between domains. For example, funding is an overarching intervention in the macro domain, influencing the implementation of favorable meso-level interventions, such as implementing a transition coordinator role. Similarly, meso-level interventions such as training for HCWs typically influence efficient communication between providers and older adults/caregivers on the micro level.

Examining international models of care transition interventions for older adults and their informal caregivers offers valuable insights that could inform and enhance Israel’s healthcare system. Several countries have implemented evidence-based approaches addressing challenges similar to those faced in Israel, providing practical frameworks that could be adapted to local cultural, social, and healthcare contexts [69]. Countries like Japan and Singapore provide valuable insights that may be considered and tailored to our context. Japan’s Long-Term Care Insurance (LTCI) system is funded by mandatory contributions from people aged 40 and older. It focuses on home care and rehabilitation, which helps shorten hospital stays and provides alternatives in the community [70]. Research shows that the LTCI has been successful in reducing hospital admissions and allowing patients to stay at home longer. It has also improved communication between hospitals and primary care providers through the care manager systems that provide older adults with hospitalization and discharge support. Care managers are vital for effectively allocating medical resources and facilitating interprofessional collaboration. They bridge information gaps, understand the living conditions and wishes of those in need, and develop care plans to provide appropriate services, emphasizing the importance of person-centered care [71–73]. Standardizing discharge templates across hospitals could help minimize errors during patient transitions and enhance continuity of care [74]. Furthermore, Japan’s housing adaptation grants help mitigate post-discharge risks [75]. Similarly, in Singapore, researchers evaluated the effectiveness of a national transitional care program for elderly adults with complex care needs and limited social support. Dedicated care coordinators coached older adult participants, helping them understand their conditions and manage their care. Findings showed reduced unplanned rehospitalizations and emergency department visits for hospitalized older adults [76]. These initiatives have the potential for adaptation in other settings, for example, Israel.

### Strengths and limitations

This study has strengths as well as limitations. First, this study used a multiple-perspective approach to gather participants’ perspectives on the research question of interest based on a recently introduced method for developing multi-level policy interventions [36]. Another strength of the study is that we recruited English- and Hebrew-speaking older adults and informal caregiver participants from different regions of Israel. Research on hospital-to-home care transitions for older adults often includes only older adults. 

While our study reached thematic saturation with our participant sample, we acknowledge several limitations. Although sample adequacy in qualitative research is often determined by saturation rather than size alone [77], our participant pool lacked representation across all relevant demographics and geographic regions. Ongoing security concerns and access restrictions in certain areas limited our recruitment capabilities, potentially excluding important perspectives. Hence, our results should be interpreted with caution as they are best understood as preliminary rather than definitive recommendations from a more representative population as a whole. Future research would benefit from broader sectoral and geographic representation to capture the full complexity of experiences across different contexts.

## Conclusions

This study presents policy recommendation categories for policymakers, healthcare professionals, and stakeholders. Our findings highlight strategies to enhance patient and caregiver engagement through personalized care plans and improved communication on a micro level while advocating for better care coordination and support services at the meso level. At the macro level, the recommendations focus on policy reforms to address systemic issues such as resource allocation and healthcare workforce capacity, aiming to facilitate smoother care transitions from hospital to home. These recommendations could tackle the multifaceted challenges associated with hospital-to-home transitions, enhancing the care experience and outcomes for older adults and their caregivers by addressing individual, organizational, and systemic issues. Future research with larger, more diverse, and representative samples is essential to validate and expand upon these initial insights. Further international studies in different contexts where these proposed policy interventions can be implemented should also be conducted.

## Supplementary Information

Supplementary material 1.

## Data Availability

Due to ethics restrictions, the datasets generated during the current study cannot be made publicly available. The research data, coding, and materials utilized in this study are available on a secure server to which the primary researcher has exclusive access.
